# Transfer efficiency and impact on disease phenotype of differing methods of gut microbiota transfer

**DOI:** 10.1038/s41598-022-24014-x

**Published:** 2022-11-15

**Authors:** Chunye Zhang, Yushu Shi, Matthew Burch, Benjamin Olthoff, Aaron C. Ericsson, Craig L. Franklin

**Affiliations:** 1 Department of Veterinary Pathobiology, University of Missouri, Columbia, MO 65201 USA; 2 Department of Statistics, University of Missouri, Columbia, MO 65201 USA; 3 Comparative Medicine Program, University of Missouri, Columbia, MO 65201 USA; 4 University of Missouri College of Veterinary Medicine, Columbia, MO 65201 USA; 5 University of Missouri Metagenomics Center, Columbia, MO 65201 USA; 6 Mutant Mouse Resource and Research Center, University of Missouri, 4011 Discovery Drive, Columbia, MO 65201 USA

**Keywords:** Genetics, Microbiology

## Abstract

To test causal relationships between complex gut microbiota (GM) and host outcomes, researchers frequently transfer GM between donor and recipient mice via embryo transfer (ET) rederivation, cross-fostering (CF), and co-housing. In this study, we assess the influence of the transfer method and the differences in baseline donor and recipient microbiota richness, on transfer efficiency. Additionally, recipient mice were subjected to DSS-induced chronic colitis to determine whether disease severity was affected by GM transfer efficiency or features within the GM. We found that the recipient’s genetic background, the baseline richness of donor and recipient GM, and the transfer method all influenced the GM transfer efficiency. Recipient genetic background and GM both had significant effects on DSS colitis severity and, unexpectedly, the transfer method was strongly associated with differential disease severity regardless of the other factors.

## Introduction

The collection of microorganisms that live in human and non-human animals’ gastrointestinal tract, known as gut microbiota (GM), contribute to health and disease^1–3^. Previous studies have shown that supplier-dependent differences in the GM significantly influence disease phenotypes in several disease models, such as the IL10^−/−^ mouse model of inflammatory bowel disease (IBD) and the Apc^+/min^ mouse model of colorectal cancer^4,5^. Experimental transfer of the GM using animal models is a popular approach to identify associations between differing GM and disease phenotypes, create a well-controlled GM environment for further investigation of underlying mechanisms, and improve the reproducibility of biomedical research using animal models^6–12^. Accumulating studies show that many factors can contribute to the variation of the GM^13–15^. In this project, we focus on different methods of GM transfer and the subsequent change of disease phenotype in mice generated via the different methods. Additionally, the influence of host genotype was investigated using embryo transfer rederivation of two substrains of C57BL/6 mice: C57BL/6 J and C57BL/6 N.

There are several commonly used methods of experimental GM transfer^16,17^. Embryo transfer (ET) using surrogate dams^18^ harboring a desired GM was considered the gold standard for these studies as it represents the most natural means of gut microbiota transfer from mother to pup. Simply co-housing (CH) GM donor and recipient mice is also commonly used in the literature^19–22^, although the presence of a mature GM in recipient mice presumably limits the completeness of GM transfer. As ET requires considerable expertise and infrastructure, cross-fostering (CF) of pups within the first 24 h of life to a surrogate dam harboring the GM of interest represents a third option to be used as a GM transfer method^16,17^. The latter shows promise as a means of GM transfer from the surrogate dam (i.e., GM donor) based on its common use to eliminate targeted bacterial pathogens^23,24^. Our hypothesis is that the different GM transfer methods differ in transfer efficiency, and result in subsequent differences in the disease phenotype of a commonly used mouse model. Specifically, we hypothesized that ET would provide complete transfer of the GM from birth dam to offspring, CH would result in the lowest transfer efficiency, and CF would be intermediate between ET and CH in transfer efficiency.

As we have previously shown that transfer of the GM between mice via repeated gastric gavage of antibiotic-treated mice is dependent on the relative richness of the donor and recipient GM, two different substrains of the commonly used C57BL/6 mouse, C57BL/6 J (B6J) and C57BL/6 N, harboring low richness and high richness GM^25^, respectively, were used as GM recipients. CD1 mice harboring the well-characterized low richness GM1 and high richness GM4 (originally derived from C57BL/6 J and C57BL/6 N mice, respectively) were used as GM donors^18^. This allowed for reciprocal transfers (i.e., high richness GM4 donors and low richness B6J recipients, and low richness GM1 donors and high richness B6N recipients) using each of the three different transfer methods. Additionally, the ET studies were performed using a fully crossed study design to determine the influence of recipient substrain genetics on the composition of the GM at adulthood. At 7 weeks of age, all recipient mice were subjected to the DSS-induced model of chronic colitis using the classical approach^26,27^. The primary metrics of disease severity were survival, percent weight change, colon length, and histological scoring of colitis severity.

## Results

### Study group nomenclature

**Figure 1:**
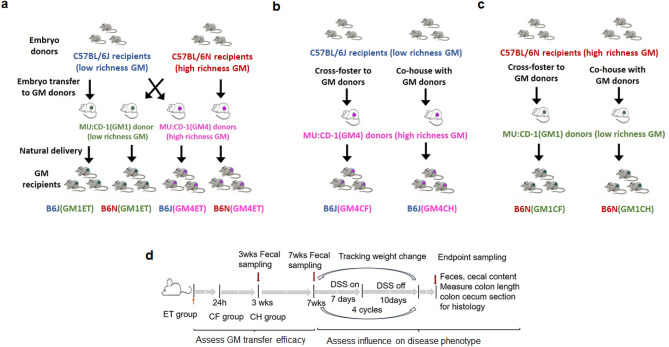
Experimental design of GM transfer studies and chronic DSS colitis. (**a)** Substrain controlled transfer of GM1 and GM4. (**b)** Transfer of GM4 using cross fostering and co-housing methods. (**c)** Transfer of GM1 using cross fostering and co-housing methods. (**d)** Timeline of DSS treatment to induce chronic colitis.

Throughout the manuscript, the different groups of mice are designated as recipient substrain (B6N or B6J) followed by the GM to be transferred (GM1 or GM4) and the method of transfer (ET, CF or CH). For example, B6N (GM4ET) represents a B6N mouse to which GM4 has been transferred using embryo transfer. The study design and schematic graph are presented in Fig. 1.

### Substrain genetics influences the GM transfer efficiency following ET

In order to first assess the influence of substrain genetics on GM transfer efficiency, the gold standard GM transfer method (ET) was used to generate four groups of recipient mice: B6J(GM1ET), B6J(GM4ET), B6N(GM1ET), and B6N(GM4ET).

**Figure 2:**
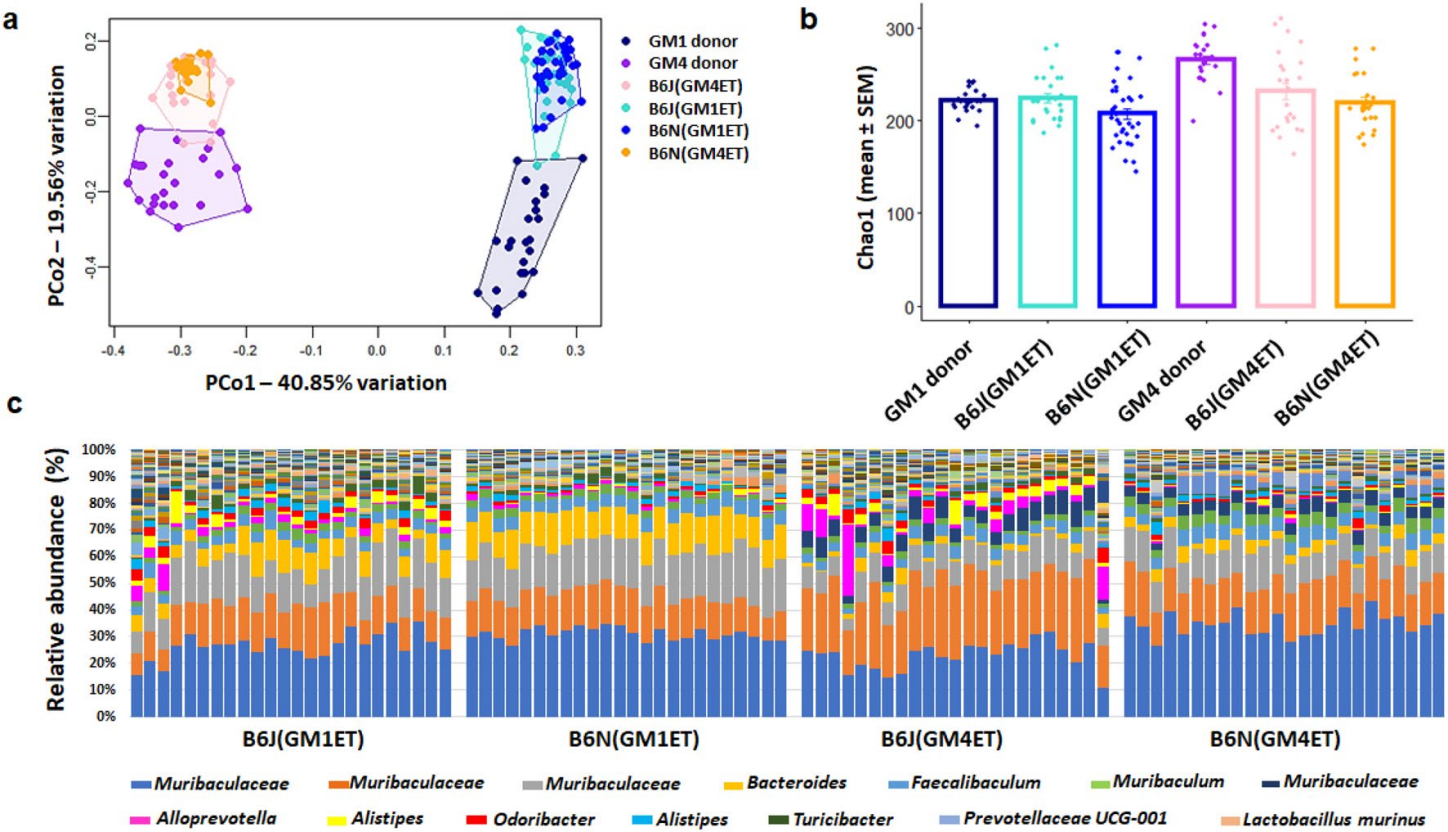
Donor GM (primary) and recipient genetics (secondary) sources of GM variability in embryo transfer derived recipient mice**. (a)**Principal Coordinate Analysis plot of OTUs from fecal samples of 7-week-old embryo transfer (ET) derived B6J and B6N mice colonized with GM1 or GM4. (GM1 vs. GM4: *p* = 0.001, F = 189.17; B6J vs. B6N: *p* = 0.001, F = 44.65, Bray–Curtis.). (**b)** Chao-1 index of 7-week-old mice generated using the ET method revealed main effects: B6J vs. B6N *p* = 6.29e-06, F = 13.00; GM1 vs. GM4: *p* = 4.07e-06, F = 22.91 as well as an interaction between recipient genotype and GM *p* = 0.0049, F = 5.51; ANOVA followed by Bonferroni adjustment for 4 pairs group comparison. (**c)** Bar chart showing differing relative abundance of all taxa detected in substrains B6J and B6N that received GM4 and GM1.

Principal coordinate analysis (PCoA) of 16S rRNA data revealed that the donor GM was the primary determinant of the offspring GM composition at 7 weeks, with substrains receiving GM1 or GM4 from their birth dam separating along PCo1 (40.85% variation) (Fig. 2a). However, PCoA also revealed significant substrain-dependent differences captured by PCo2 (19.56% variation), which were confirmed using one-way PERMANOVA (*p* = 0.001; F = 44.65, Bray–Curtis). This result indicated that the donor (i.e., biological dam) GM is the primary source of variability affecting the transfer outcome, but that differences in recipient genetics, even at the level of substrain, also affect the final GM composition.

As expected, the Chao-1 richness index was significantly different between GM1 and GM4 (Fig. 2b). Moreover, there was a significant interaction between recipient genotype and GM which may be due to heterogenous effects of GMs on different genotypes, or heterogenous effects of genotypes for different GMs. To further assess this interaction, a posthoc analysis with Bonferroni adjustment was performed for 4 comparisons revealing no significant differences between the GM1 donors and either B6J(GM1ET) or B6N(GM1ET) recipients. In contrast, there were significant differences between GM4 donors and both B6J(GM4ET) (*p* = 9.87e-03) and B6N(GM4ET) (*p* = 1.28e-06).

In addition to the expected differences between GM1 and GM4, there were differences in the patterns of relative abundance in a substrain-based manner regardless of the GM received (Fig. 2c). In other words, certain taxonomies were preferentially enriched in one substrain as compared to the other substrain, in both of the tested GMs.

In conclusion, following ET transfer, the offspring GM is highly similar to the GM from the surrogate (birth) dam in richness and composition, however the recipient genetics also subtly influence the transferred GM composition.

### Mice generated using ET differ in susceptibility to DSS-induced colitis in GM- and substrain-dependent manner

**Figure 3:**
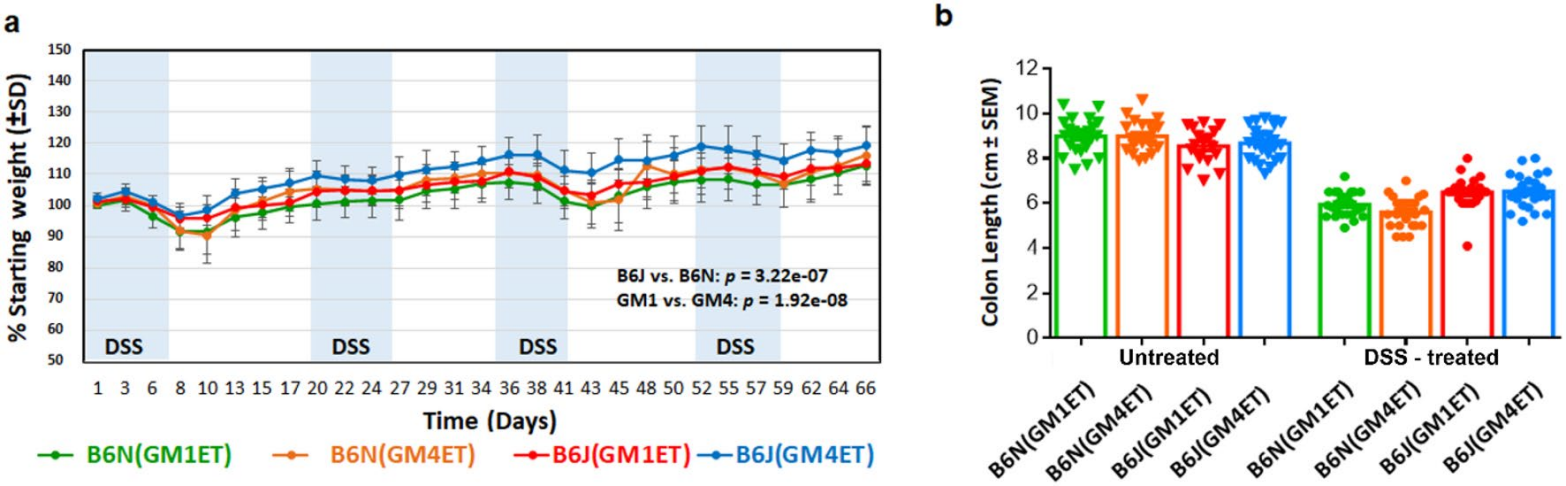
Influence of substrain genetics of recipient and the donor GM on the susceptibility to DSS-induced colitis in mice derived by embryo transfer. (**a)** Comparison of DSS-induced weight change between substrains that received GM1 and GM4 via embryo transfer. Statistical analysis revealed the significant main effects of substrain (B6J vs. B6N: *p* = 3.22e-07), GM (GM1 vs. GM4: *p* = 1.92e-08), time (*p* = 9.38e-90), and DSS treatment (*p* = 1.71e-35) with an interaction of time and DSS treatment, *p* = 1.52e-12). The longitudinal measurements of each mouse were considered. A random intercept mixed effect model with AR covariance structure was performed for statistical analysis. (**b)** Comparison of colon lengths revealed a significant main effect of treatment (*p* < 2e-16, F = 546.38). While there was not a main effect of GM (ANOVA), visual inspection of the data showed that untreated B6N mice had longer colon lengths than untreated B6J mice but shorter colon lengths after DSS treatment.

To assess the influence of different GMs and substrains on disease susceptibility, mice were subjected to a commonly used regimen of cyclical DSS administration to induce chronic, intermittent colitis. Comparison of DSS-associated effects on weight showed that B6J(GM4ET) mice experienced less weight change post-treatment compared with other ET groups (Fig. 3a). The B6N(GM1ET) group experienced greater weight change compared with other ET groups. Statistical analysis revealed that substrain (B6J vs. B6N: *p* = 3.22e-07), GM (GM1 vs. GM4: *p* = 1.92e-08), sex (*p* = 0.002), time (*p* = 9.38e-90), and DSS treatment (*p* = 1.71e-35) with interactions (time and DSS interaction, *p* = 1.52e-12) affected weight change.

Comparisons of DSS-associated colon length (Fig. 3b) showed a main effect of treatment (*p* < 2e-16, F = 546.38), confirming that DSS administration resulted in significant shortening of the colon. A trend was noted that, while untreated B6N mice had greater colon lengths compared to B6J mice, B6N mice in DSS-treatment groups, regardless of GM, showed greater colon shortening than B6J mice. Moreover, Two-Way Analysis of Variance with Student–Newman–Keuls post-hoc analysis of lesions scores demonstrated a significant main effect (*P *< 0.001) of strain, but not GM (*P *= 0.34) with the following mean lesion scores (+ /1 SEM) for each group: B6J(GM1ET) = 4.04 ± 0.69.; B6J(GM4ET) = 3.78 ± 0.18; B6N(GM1ET) = 4.39 ± 0.2; B6N(GM4ET) = 4.96 ± 0.12. A significant (*P *= 0.01) substrain x GM interaction was also found with GM4 and GM1 being significantly different (*P *< 0.05) in the B6N substrain. These findings validated the differences observed in weight change, and collectively, these results highlight the integrated influence of host genetics and GM on susceptibility to DSS-induced weight change and colitis.

### Transfer methods (ET, CF, CH) differ in transfer efficiency when transferring high richness GM4 to recipient B6J mice

Studies comparing different transfer methods were performed using CD-1 donor mice of either high richness GM4 or low richness GM1 (derived from Envigo and Jackson, respectively) and recipients from the reciprocal source.

**Figure 4:**
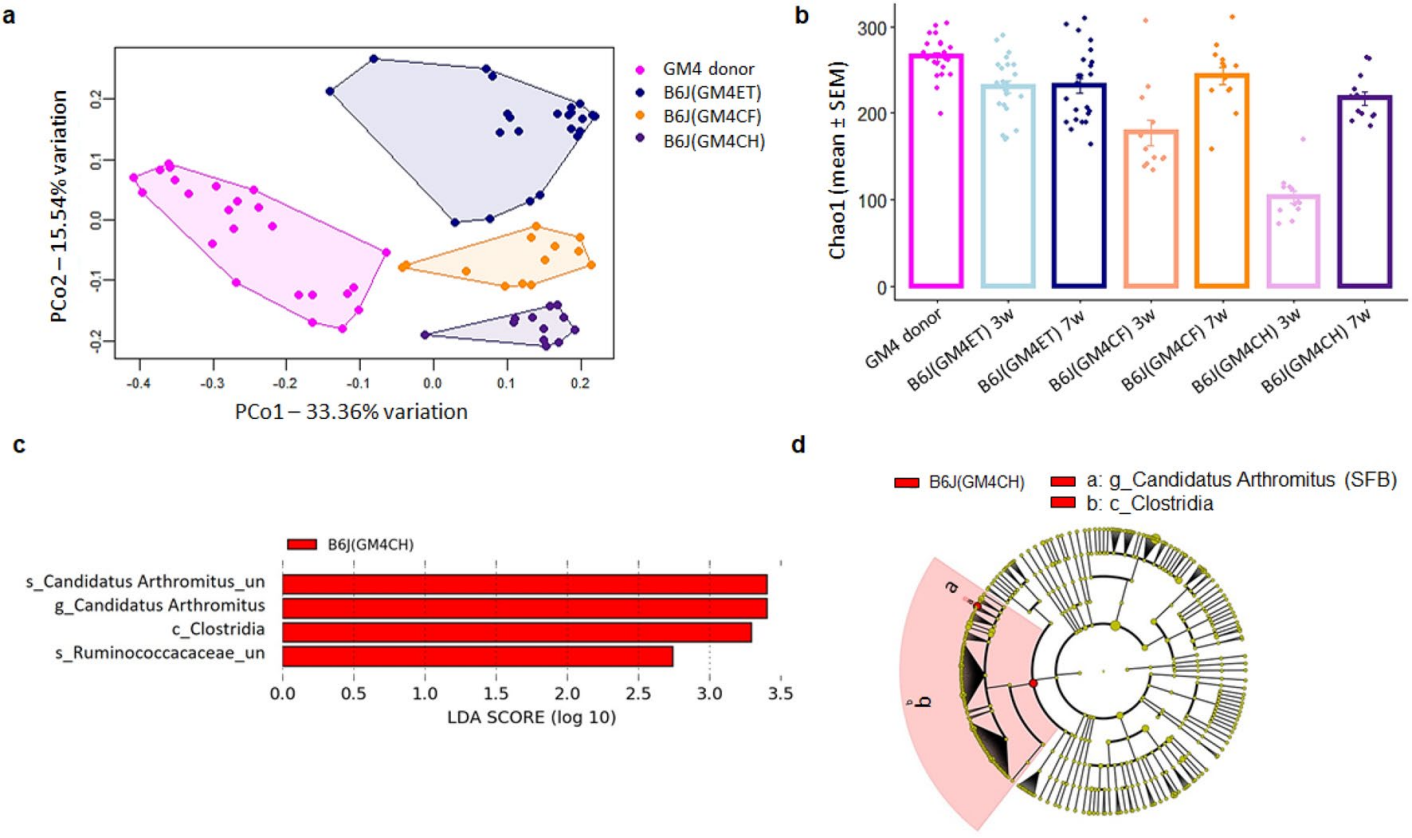
Influence of different transfer methods on transfer efficiency when transferring high richness GM4 to B6J mice. (**a)** Principal Coordinate Analysis plot of OTUs from feces of 7-week-old B6J mice receiving GM4 via embryo transfer (ET), cross fostering (CF), and co-housing (CH) as compared to their CD-1(GM4) donors showed a main effect of transfer method (*p* = 0.001; F = 21.05, Bray–Curtis). (**b)** Statistical analysis (ANOVA) of the Chao-1 index of data from the same B6J mice also showed a main effect of transfer method at both 3 weeks (*p* = 2.2e-16; F = 69.897) and 7 weeks (*p* = 6.2e-4; F = 6.49) with the difference between transfer method and donor dam increasing from CH > CF > ET at 3 weeks. (**c)** Linear discriminant analysis effect size (LEfSe) analysis and (**d**) Cladogram show compositional differences and taxa that were significantly overrepresented in each group. Cladogram circles represent the different taxonomic levels from phylum (innermost circle) to species (outermost circle). Annotation represents p_ phylum, c_ class, o_ order, f_ family, g_ genus, or s_ species from the inside to the outside.

Principal coordinate analysis of GM4 donors and B6J recipients from all three transfer methods showed a clear separation of all three recipient groups (Fig. 4a), with differences confirmed by One-way PERMANOVA (*p *= 0.001; F = 21.05, Bray–Curtis). Samples from mice in the ET and CF groups clustered closer to the donor samples than samples from recipient mice generated using the CH method. As expected, marked separation of the CH group from donor controls was also observed at 3 weeks of age (Supplementary Fig. 1A). At 3 weeks of age, the richness of GM was significantly lower than the dam’s GM in recipients from all three transfer groups (*p* = 1.5e-13, ET, *p* = 5.18e-10, CF, *p* = 0, CH). However, the difference was greatest in the co-housing group (161.75), followed by the cross-fostering group (87.17) and the embryo transfer group (34.88) (Fig. 4b). By 7 weeks of age, richness had increased in all three groups but remained significantly different from the dam in the ET (*p* = 3.9e-3) and CH (*p* = 5.2e-4) groups and was more equivalent between groups (CH = 47.58, CF = 21.75, ET = 33.0). LEfSe analysis (Fig. 4c and Fig. 4d) detected a limited number of taxa preferentially enriched at 7 weeks in B6J mice receiving GM4 via CH, relative to ET and CF, all within the class *Clostridia* and including an annotation Candidatus Arthromitus likely representing segmented filamentous bacteria (SFB). Thus, while all three methods resulted in some degree of GM transfer, the transfer efficiency of high richness GM4 into B6J mice differed between methods in transfer completeness, with ET and CF providing more complete transfer than CH.

### B6J(GM4) mice generated using different transfer methods display differential susceptibility to DSS-induced colitis

**Figure 5:**
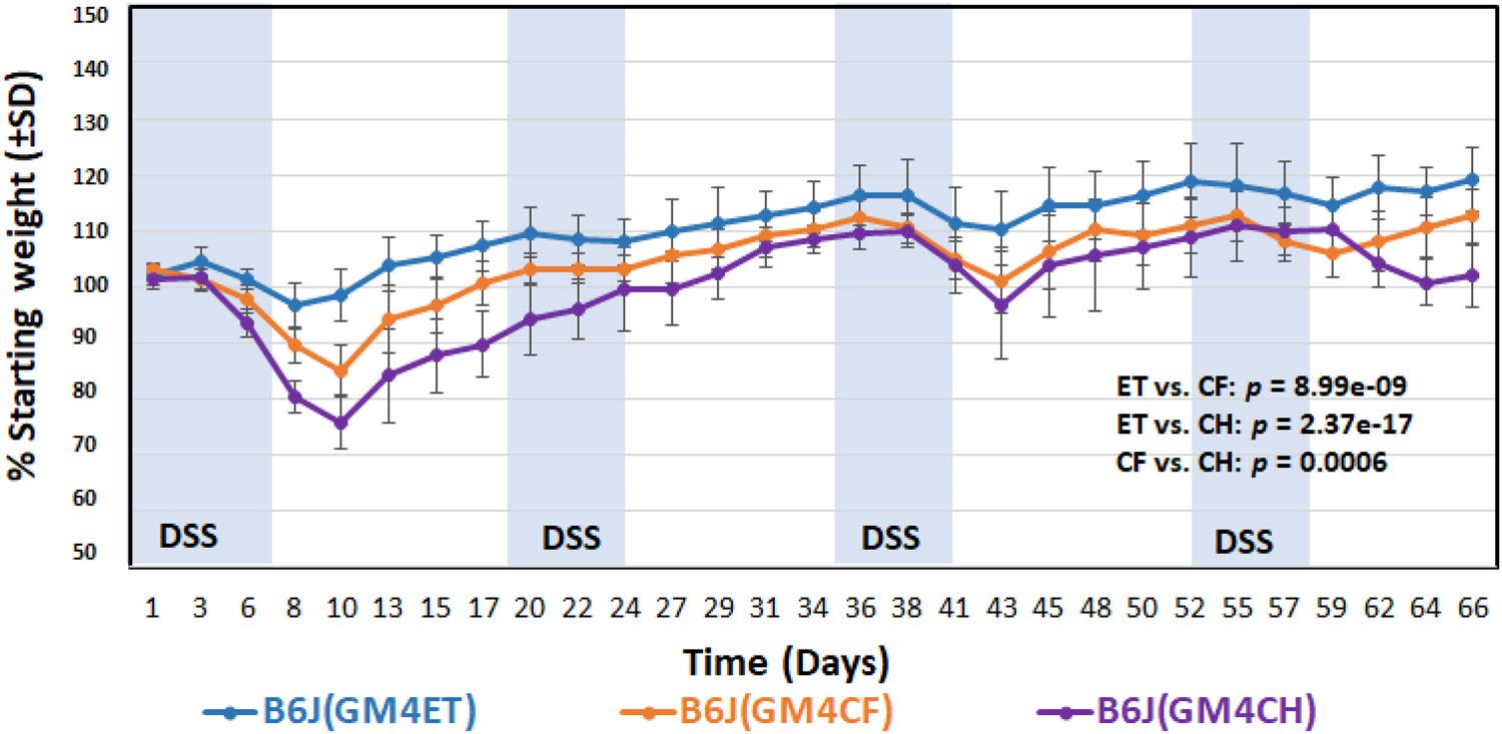
Influence of transfer method on susceptibility to DSS-induced colitis when transferring high-richness GM4 to B6J mice**.** Comparison of DSS-induced weight change between B6J mice that were recipients of GM4 using ET, CF, and CH transfer methods. Statistical analysis revealed a significant difference between transfer methods (ET vs. CH: *p* = 2.37e-17; ET vs. CF: *p* = 8.99e-09; CF vs. CH: *p* = 0.0006). There were significant effects of time (*p* = 2.99e-41), DSS treatment (*p* = 2.04e-19), and an interaction of time and DSS treatment (*p* = 5.60e-05). However, there was no significant effect of sex (*p* = 0.755); as a result, the figure shows data from both sexes. The longitudinal measurements of each mouse were considered. A random intercept mixed effect model with AR covariance structure was performed for statistical analysis.

Administration of DSS to B6J mice that received high richness GM4 via different transfer methods resulted in significant differences in weight change depending on the transfer method (Fig. 5). Specifically, the DSS-induced weight change in the CH group was significantly greater than that in ET and CF groups. However, comparison of colon lengths and lesion scores post-DSS treatment among B6J recipient mice that received GM4 using different transfer methods showed no significant difference between groups (data not shown). The differences in weight change suggest that GM4 may be less protective against disease when incompletely transferred at a later age via co-housing but the degree of protection may be minor given that no differences were seen in other measures of disease severity.

### Transfer methods differ in transfer efficiency when transferring low richness GM1 to recipient B6N mice

**Figure 6:**
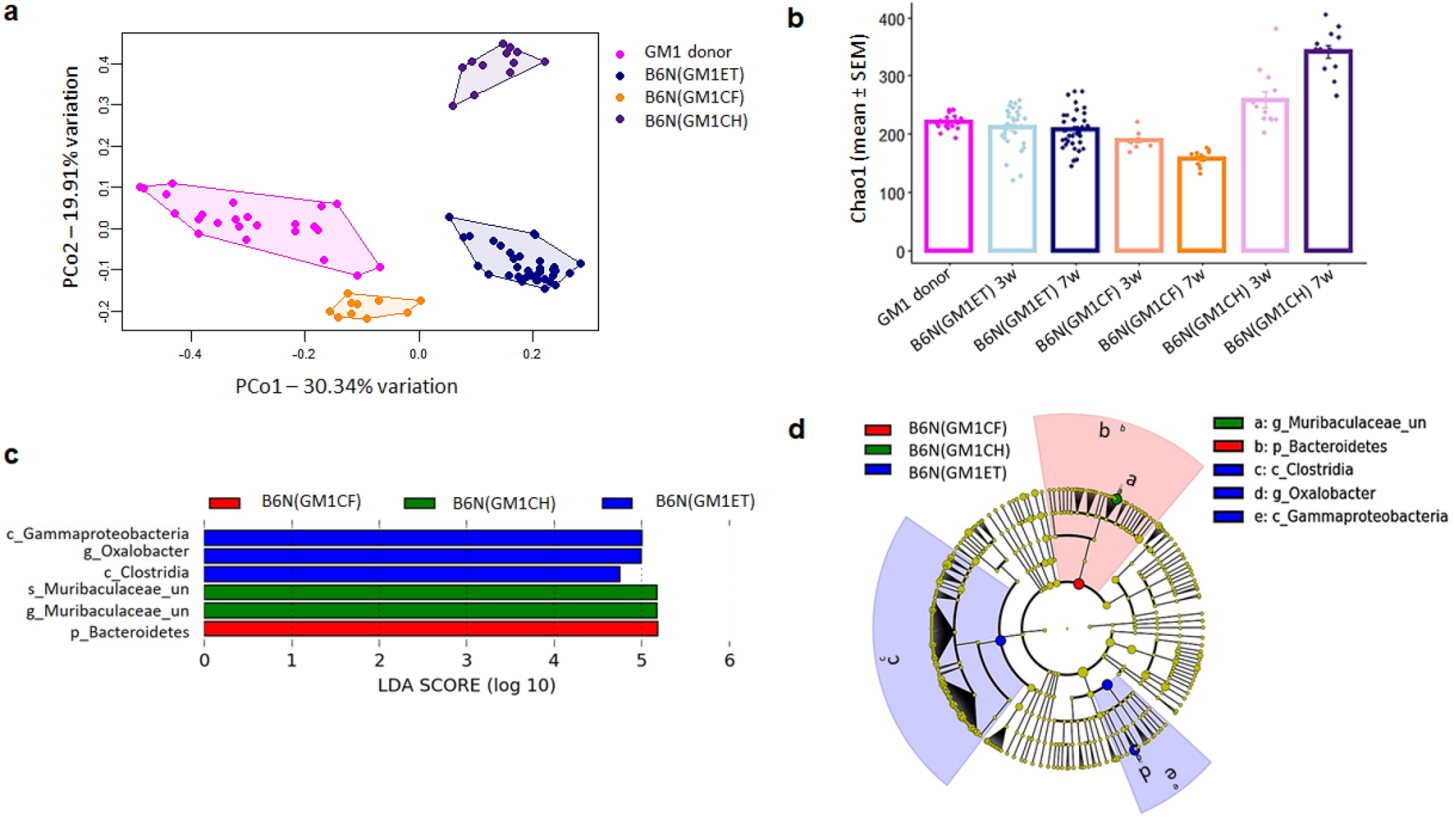
Influence of different transfer methods on transfer efficiency when transferring low richness GM1 to B6N mice**. (a)**Principal Coordinate Analysis plot of OTUs from feces of 7-week-old B6N mice receiving GM1 via embryo transfer (ET), cross fostering (CF), and co-housing (CH) as compared to their CD-1(GM1) donors showed a main effect of transfer method (*p* = 0.001; F = 44.54, Bray–Curtis). (**b**) Statistical analysis (ANOVA) of the Chao-1 index of data from the same B6N mice also showed a main effect of transfer method at both 3 weeks (*p* = 3.77e-6; F = 11.11) and 7 weeks (*p* = 2.2e-16; F = 96.85). (**c**) Linear discriminant analysis (LDA) effect size (LEfSe) analysis and (**d)** Cladogram showed compositional difference and taxa that were significantly overrepresented in each group when performing the comparison between mice groups that generated using ET, CF and CH method, B6N (GM1ET), B6N (GM1CF) and B6N (GM1CH) at 7 weeks of age. Cladogram annotation: p_ phylum, c_ class, o_ order, f_ family, g_ genus, or s_ species from inside to outside. Red, green and blue color-coded nodes stand for the species that are overrepresented in each group).

To further assess the influence of transfer method and differences in richness between donor and recipient on transfer efficiency, we transferred low richness GM1 into B6N mouse using all three methods. One-way PERMANOVA comparing donor and recipient groups showed the significant differences between donor and recipients generated using the different methods. Principal coordinate analysis of GM1 donors and B6N recipients generated using ET, CF and CH methods showed a clear separation of the CH group from the ET and CF groups (Fig. 6a), with differences confirmed by One-way PERMANOVA (*p* = 0.001; F = 41.54, Bray–Curtis). Samples from mice generated using ET and CF methods also clustered much closer to donor samples than samples from recipients generated using CH. Similarly, notable separation of the CH group from donor control was also observed at 3-weeks of age recipient mice (Supplementary Fig. 1b). Comparisons of richness (Fig. 6b) revealed that, at 3- and 7-weeks of age, the richness of samples from recipients generated using ET showed no difference from the richness of donor samples. Samples from recipients generated via CF were less rich than donor samples at 3-weeks of age and remained low at 7 weeks of age. In contrast, CH recipients harbored a significantly greater richness GM pre-transfer which remained significantly greater than that of donors following CH. In contrast to the transfer of GM4 to B6J recipients, transfer of low richness GM1 to B6N mice was less effective, particularly in the CH group (the richness of donor and recipient at 7 weeks of age: *p* = 0.000). LEfSe analysis of recipients at 7 weeks of age revealed significantly enriched taxa in mice generated using ET, CF, and CH methods (Fig. 6c and Fig. 6d). In conclusion, for both transfer directions, our results showed that the ET and CF methods resulted in greater transfer completeness and efficiency compared with the CH method.

### B6N (GM1) mice generated using different transfer methods display differential susceptibility to DSS-induced colitis

**Figure 7:**
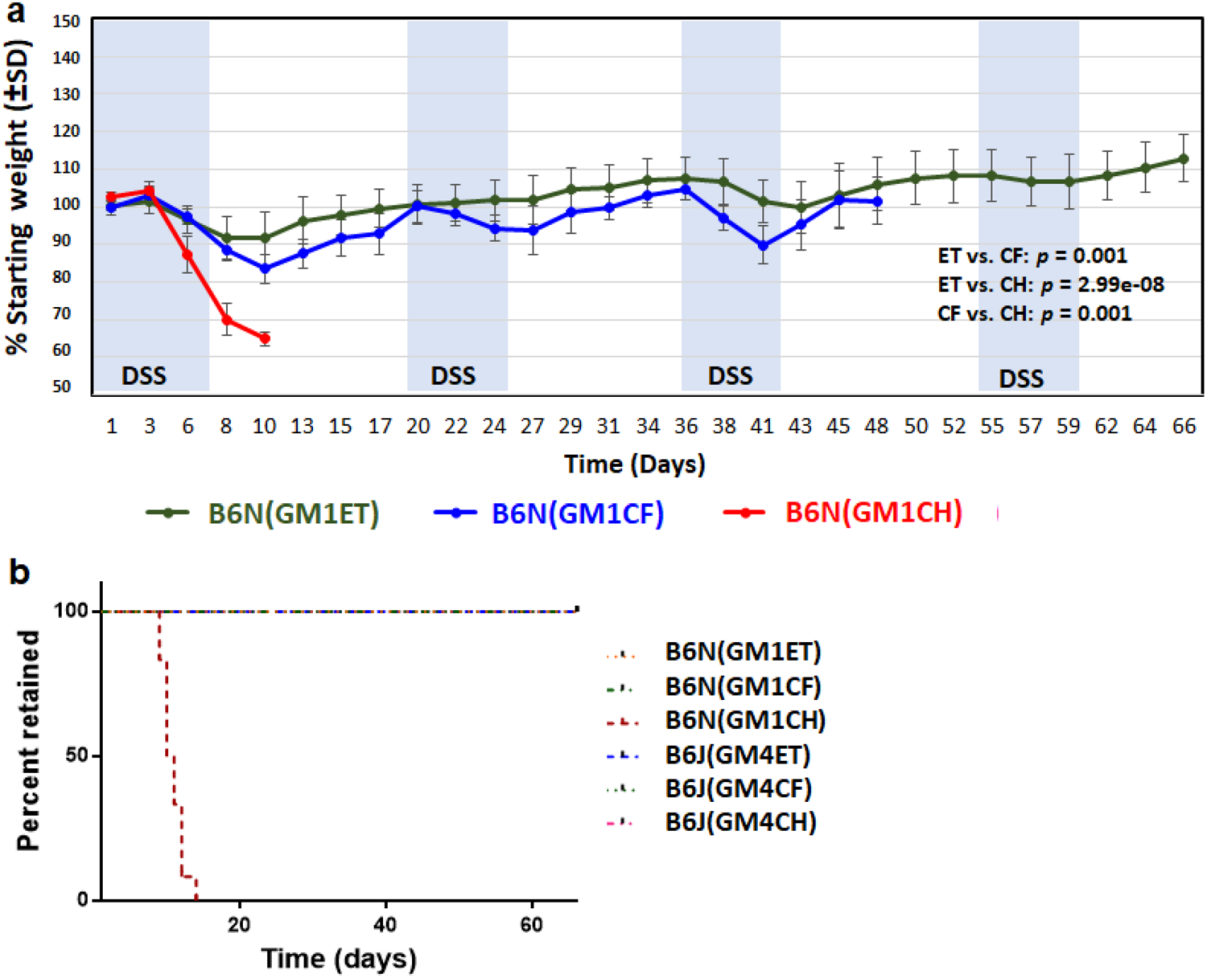
Influence of transfer method on susceptibility to DSS-induced colitis when transferring low-richness GM1 to B6N mice. (**a**) Comparison of DSS-induced weight change between recipient B6N that were recipient of GM1 using ET, CF, and CH transfer method. Statistical analysis revealed the significant difference between transfer methods (ET vs. CH: *p* = 2.99e-08; ET vs. CF: *p* = 0.001; CF vs. CH: *p* = 0.001). There were significant main effects of time (*p* = 6.66e-21), sex (*p* = 0.013), DSS treatment (*p* = 9.07e-43), and a time / DSS treatment interaction (*p* = 2.79e-27). The longitudinal measurements of each mouse were considered. Random intercept mixed effect model with AR covariance structure was performed for statistical analysis. Two-factor (time and transfer method) ANOVA. The figure shows data from both sexes. (**b**) Survival curve of B6J and B6N mice groups generated using the different transfer methods ET, CF, and CH. (Kaplan Meier survival analysis in GraphPad Prism). Analysis using a Log-rank (Mantel-Cox) test demonstrated statistical significance of this observation (*p *< 0.0001). From days 49–66, weights were inadvertently not recorded for the B6N (GM1CF) group.

Comparison of DSS-induced weight change between transfer groups paralleled that seen in B6J(GM4) mice, as mice generated using ET had significantly less weight change compared to the CF and CH groups (Fig. 7a). Remarkably, mice generated using CH experienced severe weight change (i.e., more than 20%) beginning around day 10 post-DSS treatment and were humanely euthanized according to our IACUC protocol (9587). A survival curve was used to show the rate of removal from study of B6N mice generated using CH method (Fig. 7b). Analysis using a Log-rank (Mantel-Cox) test demonstrated statistical significance of this observation (*p *< 0.0001). Colon length analysis and lesion scoring was precluded in this study as the CH group did not survive and the CF group was inadvertently euthanized prior to the ET group.

Overall, the weight change differed in the transfer groups generated using ET, CF, and CH methods. Mice in the ET group displayed significantly less weight change compared to mice generated using CF and CH, regardless of transfer direction, while mice in the CH group experienced more severe weight change, especially when attempting to transfer low richness GM1 to B6N mice already colonized with a high richness GM.

## Discussion

In recent years, modulation of gut microbiota through GM transfer has become increasingly popular in biomedical research, using multiple approaches^28–31^. The transfer of disease phenotype via GM transfer is critical for demonstrating a causative influence of the GM on a disease phenotype. Microbiota transfer is also used as a possible therapeutic approach for certain diseases.

Thus, it is essential to have a better understanding of microbiota transfer efficiency using different transfer methods and scenarios, and a sense of whether and how the different transfer methods may inherently affect the phenotype of disease models. Additionally, optimized practices for GM transfer in the research community are necessary from the perspective of reproducibility. For example, if models are found to lack reproducibility between labs, the GM can be explored as a culprit first by characterization of composition. Should differences in model phenotypes correlate with differences in GM, transfer studies can be used to begin to establish cause and effect relationships between GM and phenotype.

There are several approaches for GM transfer. ET is considered the gold standard since the pups obtain their GM through natural means including exposure to vaginal microbiota during delivery, and maternal fecal and environmental microbiota immediately after birth and during nursing and maternal care. Cross fostering of pups onto GM donor mice within the first 24 h of life represents a cost-effective alternative to ET. Offspring GM is populated by the surrogate (donor) maternal fecal and environmental microbiota but is exposed to the vaginal microbiota of the birth mother, rather than the surrogate dam^16^. Nursing and maternal care likely play a critical role in shaping the recipient microbiota in both ET and CF-derived offspring^22^ and the presence of littermates may help to amplify colonization of offspring. Inclusion of pups naturally born to the surrogate dam may also facilitate some sharing of maternal vaginal microbiota to their fostered littermates.

In contrast, co-housing of weanling recipient mice with donor mice occurs after establishment of the offspring GM and ignores developmental influences of the transferred GM on the recipient. Despite this shortcoming, co-housing is a commonly used method to normalize the microbiota between mice due to its simplicity^22^. However, it does have limitations, as demonstrated by the current study and other research teams^20,22^.

While embryo transfer results in a natural vertical GM transfer between dam and offspring, this method is costly and requires substantial expertise and infrastructure, making it an impractical method for most labs. Alternatively, the current study shows that cross-fostering results in similar transfer efficiency when compared to ET. This method is advantageous over ET because of ease of use and low cost^12,16–18^.

Co-housing as a transfer method produced mixed results. While CH resulted in less complete transfer of the rich GM4 to recipient B6J mice when compared to ET or CF, transfer was nonetheless partially effective whereas the CH-mediated transfer of the sparse GM1 to B6N recipients resulted in a hybrid recipient profile that was actually more dissimilar to donor mice than the pre-transfer profiles. These data are in agreement with previous findings showing that the successful transfer of GM to antibiotic-treated mice via repeated intra-gastric gavage is dependent on the starting relative richness of donor and recipient GMs^25^.

Co-housing also resulted in exacerbation of DSS disease severity as evidenced by increased weight change in the B6J(GM4CH) group and marked weight change leading to removal from study in the B6N(GM1CH) group. The reasons for this are unknown, but may be related to compounding weaning stress with the stress of being placed in a new cage with strange donor mice. This may result in sufficient stress to modulate the immune system and exacerbate inflammation. However, this seems unlikely as DSS was not administered until mice were seven weeks of age and had been with the donor mice for a full month. The increased disease severity (as measured via weight change) observed in B6J(GM4CH) mice relative to B6J(GM4ET) and B6JGM4CF is intuitive in the context of decreased efficiency of CH-mediated transfer of the rich GM4 shown to be protective in the fully crossed ET studies. However, the reciprocal series of transfers yielded unexpected results as the CH-mediated transfer of GM1 (shown to confer increased disease severity) to B6N, resulted in extremely poor transfer efficiency by seven weeks of age, and yet disease was severely exacerbated. We speculate that there may be differences between B6J(GM4CH) and B6N(GM1CH) mice in the antigen burden placed on the immune system due to DSS administration. B6J mice housed with GM4 donors at weaning are readily colonized with the donor GM due to the inherent difference in richness (donor > recipient) and coprophagy, and development of tolerance to those bacteria over the following four weeks may occur before administration of DSS. In contrast, B6N recipient mice may be, at least partially, resistant to colonization with the donor GM due to the inverse relationship in richness (donor < recipient), despite coprophagy. Lacking full tolerance to the GM, perhaps the immune system of B6N(GM1CH) mice is exposed to a greater number of previously unrecognized bacteria following DSS exposure and ulceration of the mucosa. While additional experiments are needed to answer those questions, the current findings provide compelling evidence that the method of transfer and relationship between donor and recipient in starting GM richness can significantly influence both transfer efficiency and model phenotypes.

These findings highlight the need for appropriate controls in studies wherein the GM is experimentally transferred, particularly using CH. Such controls could include transfer of irrelevant GM profiles not associated with phenotypic changes, transfer of the target GM using multiple methods, or simply second-generation mice born to recipient mice. The absence of such controls makes interpretation of experimental outcomes difficult. Surprisingly, we were unable to identify any controlled studies wherein different transfer methods were directly compared.

Lastly, our data demonstrate a significant effect of recipient genetics, even at the level of substrain, on the final GM profile when the GM is transferred via ET. Org et al*.* previously demonstrated host genetic control of the GM and identified loci associated with the relative abundance of certain taxa^32^. Our data provide similar evidence of such loci differing between substrains, as certain taxa are enriched in B6J recipients regardless of the GM. These findings underscore the multi-layered influence of host genetics and GM composition on model outcomes, and again emphasize the necessity of appropriate controls.

In conclusion, our results demonstrate that both the transfer method and transfer direction influence experimental GM transfer efficiency. ET showed the highest transfer efficiency, while the CF method, with the advantage of lower cost and complexity compared to ET, provided a possible viable alternative option for GM transfer studies where high efficiency is desired. The CH method was particularly problematic when attempting to transfer a relatively sparse GM to a recipient with a richer starting GM and collectively, our results suggest that CH should be carefully considered when used as a GM transfer approach, and only in conjunction with the appropriate controls.

## Materials and methods

### Mice

Two colonies of mice were used as GM donor mice: MU:CD-1 (CD-1) mice harboring a standard complex low richness microbiota (GM1) originating from B6J mice (the Jackson Laboratory, Bar Harbor, ME), and CD-1 mice harboring relatively high richness GM4 originating from B6NHsd (B6N) mice (Envigo, Indianapolis, IN)^18^. Separate colonies of CD-1 mice harboring those two GMs have been maintained at our facility for over 35 generations, using a rotational breeding scheme and annual introduction of external CD-1 genetics via ET to maintain allelic heterozygosity in each colony. B6J and B6N mice (offspring of mice supplied by the respective supplier) were used as GM recipient mice. GM transfers were then carried out via 1) breeding of recipient mice, collection of embryos, and surgical embryo transfer to pseudopregnant GM donor dams; 2) breeding of recipient mice and fostering of neonatal pups (< 12 h) on to surrogate GM donor dams nursing newborn litters; or 3) timed breeding of GM recipient and donor mice, followed by co-housing in a 1:1 ratio beginning at weaning (21d). These three methods resulted in recipient mice that were exposed to the donor GM at embryonic, immediate post-natal, or weaning stages of life (Fig. 1).

All mice were housed in the AAALAC International-accredited Discovery Ridge vivarium in micro-isolator cages on ventilated racks (Thoren Caging Systems Inc., Hazelton, PA). Ad libitum supply of irradiated 5058 (breeder) or 5053 (maintenance) chow (LabDiet, St. Louis, MO) and acidified autoclaved water were provided. All animal experiments were approved by the University of Missouri Institutional Animal Care and Use Committee (IACUC protocol 9587) and conducted in compliance with the Guide for the Care and Use of Laboratory Animals and the ARRIVE guidelines.

### Embryo transfer method (ET)

For this group, B6J and B6N mice, obtained directly from the Jackson Laboratory and Envigo respectively, were bred, and embryos were collected at the two-cell stage and surgically transferred to pseudopregnant CD-1 surrogate dams. Embryo collection and transfer were performed following the previously described procedure^18^. Briefly, B6J and B6N embryo donor mice were serially injected with luteinizing hormone-releasing hormone and gonadotropins for estrus synchronization. On day 4, B6J and B6N embryo donor mice were mated to intact males to generate embryos. GM donor CD-1 surrogate dams were mated to vasectomized stud males to induce pseudopregnancy. Four days later, embryos were collected and surgically transferred to plug-positive CD-1 surrogate dams. All procedures were performed at the MU Mutant Mouse Resource and Research Center.

A fully crossed study design was used for this arm of the study, with B6J and B6N embryos each being transferred to CD-1 surrogate dams harboring low richness GM1 or high richness GM4, and resulting in four different sub-groups using the embryo transfer method: B6J (GM4ET, *n* = 24), B6N (GM1ET, *n* = 24), B6J (GM1ET, *n* = 24), and B6N (GM4ET, *n* = 24). Both female and male mice were included in the experiment design.

### Cross-foster transfer method (CF)

B6J and B6N mice, obtained directly from the suppliers, were bred to generate pups for use as recipient mice. Pups were reciprocally cross-fostered to CD-1 GM donor mice harboring high richness GM4 or low richness GM1, respectively, within 24 h after birth to generate two transfer sub-groups with opposite transfer direction. B6J (GM4CF, *n* = 13) and B6N (GM1CF, *n* = 11). For these studies, CD-1 females were bred to CD-1 males two days prior to breeding of B6 mice to ensure that CD-1 surrogate dams had litters prior to B6 parturition. this ensured that B6 pups could be transferred within 24 h after birth to a foster-ready mother. Approximately three CD-1 mice/litter were kept and included in fostered litters in order to prevent cannibalism and further facilitate GM transfer. Extra pups that were not used for the study from donor or recipient were euthanized (euthanasia is done humanely) by CO_2_ asphyxiation according to protocol.

### Co-housing transfer method (CH)

B6J and B6N mice, obtained directly from the suppliers, were bred to generate recipient mice. At 21 days of age, recipient mice were weaned and reciprocally co-housed with weanling CD-1 donor harboring high richness GM4 or low richness GM1, respectively, with two donor and two recipient mice per cage, to generate two sub-groups, B6J (GM4CH, *n* = 12) and B6N (GM1CH, *n* = 12). Only females were used in the CH study to avoid the inter-male aggression among non-littermates when cohoused at weaning.

## DSS administration

At seven weeks of age, all recipient mice received freshly prepared 2.5% DSS (MP Biomedicals, mol. wt. 36–50 kDa) in their drinking water for 7 days, followed by 10 days of DSS-free drinking water, and this was repeated for four cycles to induce chronic, relapsing inflammation**.** During the DSS-treatment, the weight change was tracked every other day on an individual basis. At the end of the 4th cycle, all mice were humanely euthanized for sample collection. During the study, any mouse that lost more than 20% of its initial body weight or exhibited clinic signs such as dehydration or lethargy was humanely euthanized.

### Sample collection

At 3 and 7 weeks of age, mice were placed in a sterile autoclaved cage and allowed to defecate after which 2–3 fecal pellets were collected. After DSS treatment, mice were humanely euthanized and endpoint feces and cecal contents were collected for GM analysis. The colon length was carefully measured from the cecocolonic junction to the rectum. The colon was rinsed with 10% neutral buffered formalin, divided into three pieces and arranged in the histology cassette, followed by fixation in formalin in preparation for paraffin embedding. Fecal samples were also collected from ET recipient dams, CF surrogate dams, and all CD-1 mice used in CH experiments.

### DNA extraction

The QIAamp PowerFecal DNA Kit was used for isolation of DNA from fecal samples. Quantification of extracted DNA was performed using Qubit® 2.0 Fluorometer and Qubit dsDNA BR assay (Invitrogen) following the manufacturer's protocol.

### 16S rRNA gene library preparation and sequencing

The V4 region of the 16S rRNA gene was amplified using the U515F/806R primers^33^, to generate dual-indexed amplicon libraries which were pooled for sequencing using the Illumina MiSeq instrument and V2 chemistry with 2 × 250 bp paired-end reads (coverage approaching 100,000 reads per sample) at the University of Missouri DNA Core facility.

### Informatics analysis

DNA sequences were assembled and annotated at the MU Bioinformatics and Analytics Core facility. Primers were designed to match the 5' ends of the forward and reverse reads. Cutadapt^34^ (version 2.6; https://github.com/marcelm/cutadapt) was used to remove the primer from the 5' end of the forward read. If found, the reverse complement of the primer to the reverse read was then removed from the forward read as were all bases downstream. Thus, a forward read could be trimmed at both ends if the insert was shorter than the amplicon length. The same approach was used on the reverse read, but with the primers in the opposite roles. Read pairs were rejected if one read or the other did not match a 5' primer, and an error-rate of 0.1 was allowed. Two passes were made over each read to ensure removal of the second primer. A minimal overlap of three bp with the 3' end of the primer sequence was required for removal. The QIIME2^35^ DADA2^36^ plugin (version 1.10.0) was used to denoise, de-replicate, and count ASVs (amplicon sequence variants), incorporating the following parameters: 1) forward and reverse reads were truncated to 150 bases, 2) forward and reverse reads with number of expected errors higher than 2.0 were discarded, and 3) Chimeras were detected using the "consensus" method and removed. R version 3.5.1 and Biom version 2.1.7 were used in QIIME2. Taxonomies were assigned to final sequences using the Silva.v132^37^ database, using the classify-sklearn procedure.

### Statistical analysis

Analytical processing of 16S rRNA gene sequences such as trimming, screening and aligning were performed by the University of Missouri Informatics Research Core Facility. Statistical analyses were performed using R software. Principal Coordinate Analysis (PCoA) plots were generated using operational taxonomic units. Permutational multivariate analysis of variance (PERMANOVA) based on Bray–Curtis distances was used to compare the compositional differences between different transfer groups. ANOVA tests were used for comparing alpha diversities in terms of chao 1 richness between groups, followed by t-tests with Bonferroni adjustment if ANOVA tests were significant. To consider longitudinal measurements for each mouse in the analysis of DSS-induced weight change, we performed random intercept mixed effect model, where gene type, GM type, gene GM interaction, sex, time, DSS or not (binary) and time DSS interaction were considered as fixed effects and AR covariance structure correlation was assumed between measurements, implying observations that were far away in time were less correlated than observations close by. Because the interaction between gene and GM was not significant, it was removed from the final model. Analysis of colon length (Fig. 4B, Fig. 6B) was performed using ANOVA for 3-week and 7-week data separately. We implemented Dunnett tests comparing each group with the dam for post hoc analysis if the ANOVA tests were significant. A p value of less than 0.05 was used as statistically significant for all analyses.

### Histology examination

All slides of GI tissues were trimmed, embedded, and sectioned by the histology services of IDEXX BioAnalytics. Histopathological examination was performed by an experienced rodent pathologist boarded by the American College of Laboratory Animal Medicine in a blinded fashion to assess the development of chronic colitis after treatment. The entire colon was evaluated, and lesions were scored on a 1–6 scale based on the extent of lesion development (i.e., percent colon affected). Lesions were typical of those seen with chronic DSS treatment and consisted of ulceration with associated suppurative inflammation mucosal hyperplasia (both adjected to and independent of ulcers, foci of fibrosis suggestive of ulcer repair and hyperplasia of mucosal associated lymphoid tissue (the latter was not considered in assessment of extent of lesion development.

## Supplementary Information

Supplementary Information.

## Data Availability

All sequence data presented in the current manuscript are available in the NCBI Sequence Read Archive under BioProject ID PRJNA694463. The data supporting the results are available in the figures of this paper.

## References

[1] Kåhrström CT, Pariente N, Weiss U (2016). Intestinal microbiota in health and disease. Nature.

[2] Cho I, Blaser MJ (2012). The human microbiome: At the interface of health and disease. Nat. Rev. Genet..

[3] Clemente JC, Ursell LK, Parfrey LW, Knight R (2012). The impact of the gut microbiota on human health: An integrative view. Cell.

[4] Hart ML, Ericsson AC, Franklin CL (2017). Differing complex microbiota alter disease severity of the IL-10(−/−) mouse model of inflammatory bowel disease. Front. Microbiol..

[5] Moskowitz JE, Andreatta F, Amos-Landgraf J (2019). The gut microbiota modulates differential adenoma suppression by B6/J and B6/N genetic backgrounds in Apc(Min) mice. Mamm. Genome..

[6] Quigley EMM, Gajula P (2020). Recent advances in modulating the microbiome. F1000Res.

[7] Jain N (2020). The need for personalized approaches to microbiome modulation. Front. Public Health.

[8] Moskowitz JE (2020). Integration of genomics, metagenomics, and metabolomics to identify interplay between susceptibility alleles and microbiota in adenoma initiation. BMC Cancer.

[9] Schmidt TSB, Raes J, Bork P (2018). The human gut microbiome: From association to modulation. Cell.

[10] Kang DW (2019). Long-term benefit of microbiota transfer therapy on autism symptoms and gut microbiota. Sci. Rep..

[11] Nichols RG, Peters JM, Patterson AD (2019). Interplay between the host, the human microbiome, and drug metabolism. Hum. Genomics.

[12] Franklin CL, Ericsson AC (2017). Microbiota and reproducibility of rodent models. Lab Anim..

[13] Ericsson AC (2015). Effects of vendor and genetic background on the composition of the fecal microbiota of inbred mice. PLoS ONE.

[14] Ericsson AC (2018). The influence of caging, bedding, and diet on the composition of the microbiota in different regions of the mouse gut. Sci. Rep..

[15] Hasan N, Yang H (2019). Factors affecting the composition of the gut microbiota, and its modulation. PeerJ.

[16] Franklin CL, Ericsson AC (2020). Complex microbiota in laboratory rodents: Management considerations. ILAR J..

[17] Ericsson AC, Franklin CL (2015). Manipulating the gut microbiota: Methods and challenges. ILAR J..

[18] Hart ML (2018). Development of outbred CD1 mouse colonies with distinct standardized gut microbiota profiles for use in complex microbiota targeted studies. Sci. Rep..

[19] Neff EP (2019). Littermate wanted: Standardizing mouse gut microbiota requires more than cohousing. Lab Anim..

[20] Robertson SJ (2019). Comparison of co-housing and littermate methods for microbiota standardization in mouse models. Cell Rep..

[21] Laukens D, Brinkman BM, Raes J, De Vos M, Vandenabeele P (2016). Heterogeneity of the gut microbiome in mice: Guidelines for optimizing experimental design. FEMS Microbiol. Rev..

[22] Caruso R, Ono M, Bunker ME, Núñez G, Inohara N (2019). Dynamic and asymmetric changes of the microbial communities after cohousing in laboratory mice. Cell Rep..

[23] Artwohl JE, Purcell JE, Fortman JD (2008). The use of cross-foster rederivation to eliminate murine norovirus, Helicobacter spp., and murine hepatitis virus from a mouse colony. J. Am. Assoc. Lab. Anim. Sci..

[24] Clark SE, Purcell JE, Bi X, Fortman JD (2017). Cross-foster rederivation compared with antibiotic administration in the drinking water to eradicate bordetella pseudohinzii. J. Am. Assoc. Lab. Anim. Sci..

[25] Ericsson AC, Personett AR, Turner G, Dorfmeyer RA, Franklin CL (2017). Variable colonization after reciprocal fecal microbiota transfer between mice with low and high richness microbiota. Front. Microbiol..

[26] Chassaing B, Aitken JD, Malleshappa M, Vijay-Kumar M (2014). Dextran sulfate sodium (DSS)-induced colitis in mice. Curr. Protoc. Immunol..

[27] Perše M, Cerar A (2012). Dextran sodium sulphate colitis mouse model: Traps and tricks. J. Biomed Biotechnol..

[28] Collins SM, Kassam Z, Bercik P (2013). The adoptive transfer of behavioral phenotype via the intestinal microbiota: Experimental evidence and clinical implications. Curr. Opin. Microbiol..

[29] Vrieze A (2012). Transfer of intestinal microbiota from lean donors increases insulin sensitivity in individuals with metabolic syndrome. Gastroenterology.

[30] Kellermayer R (2019). Fecal microbiota transplantation: Great potential with many challenges. Transl. Gastroenterol. Hepatol..

[31] Ellekilde M (2014). Transfer of gut microbiota from lean and obese mice to antibiotic-treated mice. Sci. Rep..

[32] Org E (2015). Genetic and environmental control of host-gut microbiota interactions. Genome Res..

[33] Caporaso JG (2011). Global patterns of 16S rRNA diversity at a depth of millions of sequences per sample. Proc. Natl. Acad. Sci. USA.

[34] Martin M (2011). Cutadapt removes adapter sequences from high-throughput sequencing reads. EMBnet. J..

[35] Bolyen E (2019). Reproducible, interactive, scalable and extensible microbiome data science using QIIME 2. Nat. Biotechnol..

[36] Callahan BJ (2016). DADA2: High-resolution sample inference from Illumina amplicon data. Nat. Methods.

[37] Pruesse E (2007). SILVA: A comprehensive online resource for quality checked and aligned ribosomal RNA sequence data compatible with ARB. Nucleic Acids Res..

