# Supplementary material for: Transfer efficiency and impact on disease phenotype of differing methods of gut microbiota transfer
Source: Sci Rep. 2022 Nov 15;12:19621. doi: 10.1038/s41598-022-24014-x (PMC9666633; doi:10.1038/s41598-022-24014-x)
Supplement: Supplementary file 1 — Supplementary Information. [file 41598_2022_24014_MOESM1_ESM.docx]

**Transfer efficiency and impact on disease phenotype of differing methods of gut microbiome transfer**

Chunye Zhang^1^, Yushu Shi^2^, Matthew Burch^1^, Benjamin Olthoff^3^, Aaron C. Ericsson^1,4,5,6,^ *, Craig L. Franklin^1,4,5,6,^ *

^1^ Department of Veterinary Pathobiology, University of Missouri, Columbia, MO 65201, USA.

^2^ Department of Statistics, University of Missouri, Columbia, MO 65201, USA.

^3^ Comparative Medicine Program, University of Missouri, Columbia, MO 65201, USA.

^4^ University of Missouri College of Veterinary Medicine, Columbia, MO 65201, USA.

^5^ University of Missouri Metagenomics Center, Columbia, MO 65201, USA.

^6^ Mutant Mouse Resource and Research Center, University of Missouri, 4011 Discovery Drive, Columbia, MO 65201, USA.

*Co-corresponding authors: Dr. Aaron Ericsson, [ericssona@missouri.edu](about:blank). Dr. Craig Franklin, [FranklinC@missouri.edu](about:blank)

**SUPPLEMENTARY DATA**

# Supplementary Figure 1. Principal Coordinate Analysis plot of fecal samples from 3-week-old mice following GM transfer by different methods. a Following transfer of high richness GM4 to B6J mice via embryo transfer (ET), cross fostering (CF), and co-housing (CH), fecal samples of 3-week-old mice were compared to CD-1(GM4) donors. This analysis revealed a main effect of transfer method (*p* = 0.0001; F = 38.51, Bray-Curtis) with marked separation of the CH group; b Following transfer of low richness GM1 to B6N mice via embryo transfer (ET), cross fostering (CF), and co-housing (CH) fecal samples of 3-week-old mice were compared to CD-1(GM1) donors. This analysis also revealed a main effect of transfer method (*p* = 0.0001; F = 16.43, Bray-Curtis) with marked separation of the CH group.

**
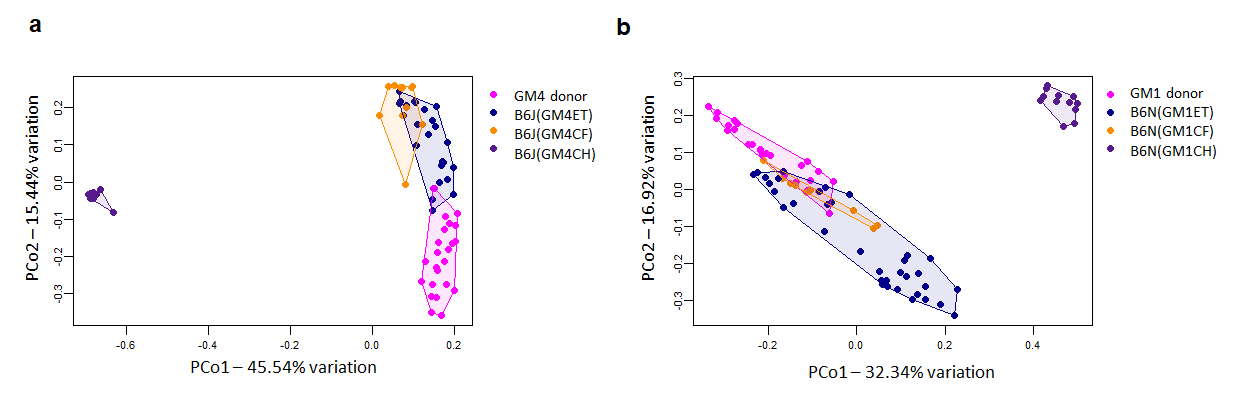
**
